# Neuropeptides: The Evergreen Jack‐of‐All‐Trades in Neuronal Circuit Development and Regulation

**DOI:** 10.1002/bies.202400238

**Published:** 2024-12-26

**Authors:** Zsofia Hevesi, Tomas Hökfelt, Tibor Harkany

**Affiliations:** ^1^ Department of Molecular Neurosciences Center for Brain Research Medical University of Vienna Vienna Austria; ^2^ Department of Neuroscience Karolinska Institutet Solna Sweden

## Abstract

Neuropeptides are key modulators of adult neurocircuits, balancing their sensitivity to both excitation and inhibition, and fine‐tuning fast neurotransmitter action under physiological conditions. Here, we reason that transient increases in neuropeptide availability and action exist during brain development for synapse maturation, selection, and maintenance. We discuss fundamental concepts of neuropeptide signaling at G protein‐coupled receptors (GPCRs), with a particular focus on how signaling at neuropeptide GPCRs could underpin neuronal morphogenesis. We use galanin, a 29/30 amino acid‐long neuropeptide, as an example for its retrograde release from the dendrites of thalamic neurons to impact the selection and wiring of sensory afferents originating at the trigeminal nucleus through galanin receptor 1 (GalR_1_) engagement. Thus, we suggest novel roles for neuropeptides, expressed transiently or permanently during both pre‐ and postnatal neuronal circuit development, with potentially life‐long effects on circuit layout and ensuing behavioral operations.

## Graphical abstract

Neuropeptides provide an efficient alternative to fast neurotransmitters for synapses neurotransmission. Here, a perspective on the roles, eventual redundancy, and receptor‐level signal diversity of neuropeptides during neurodevelopment is provided through galanin as an example. Image shows thalamic neurons in Gal‐Cre:Ai9 mice. Courtesy of J. Bakker.

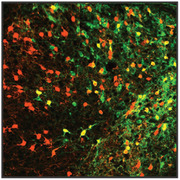

## 1 Introduction

The mammalian brain is the most complex natural system known to mankind. Although our knowledge of brain structure and function is rapidly expanding, key mechanistic details, particularly related to intercellular messengers that affect synaptic plasticity, remain incomplete. It is increasingly accepted that it is not the sheer number of neurons per se that defines complexity, operational variability, and adaptive flexibility, but their intrinsic heterogeneity, including their many modes of communication. The molecules that chiefly define intercellular signaling in the nervous system are the fast neurotransmitters (e.g., acetylcholine,  γ‐aminobutyric acid [GABA], and glutamate), which are chemical messengers delivering information from one nerve cell to another at the millisecond scale. Until recently, fast neurotransmitters were considered as the hubs of activity‐dependent circuit establishment and communication. In turn, only a modulatory or “helper” role was attributed to the group of neuroactive substances (including the >100 neuropeptides, cytokines, and lipids). Based on recent results, an alternative concept is emerging wherein neuropeptides are assigned to roles quasi‐equal in efficacy, diversity, and abundance to those of fast neurotransmitters in many organizational and operational processes during circuit development [1, 2] and the plasticity of mature synapses [3], respectively. Here, we outline a theoretical framework for combinatorial neuropeptide action through receptor redundancy and synapse specificity, using galanin as an example in both developmental and adult contexts.

## 2 Phylogenetic Diversity of Neuropeptides in Non‐Mammalian and Mammalian Organisms

Neuropeptide/receptor co‐evolution likely dates to ancient organisms that were ancestral to both *Protostomes* and *Deuterostomes* >700 million years ago [4, 5]. The emergence of the most primitive nervous systems coincided with neuropeptide signaling being the abundant and likely only form of intercellular communication, as suggested by the absence of fast neurotransmitters, gasotransmitters, catecholamines, and other biogenic amines (except serotonin) [6, 7] in, for example, cnidarians (jellyfish, corals) [8, 9, 10, 11]. However, the fast actions of neuropeptides in these species are dependent on their binding to neuropeptide‐gated ion channels [12], which were replaced by G protein‐coupled receptors (GPCRs) co‐evolving with their cognate neuropeptides in organisms with more complex nervous systems.

**FIGURE 1:**
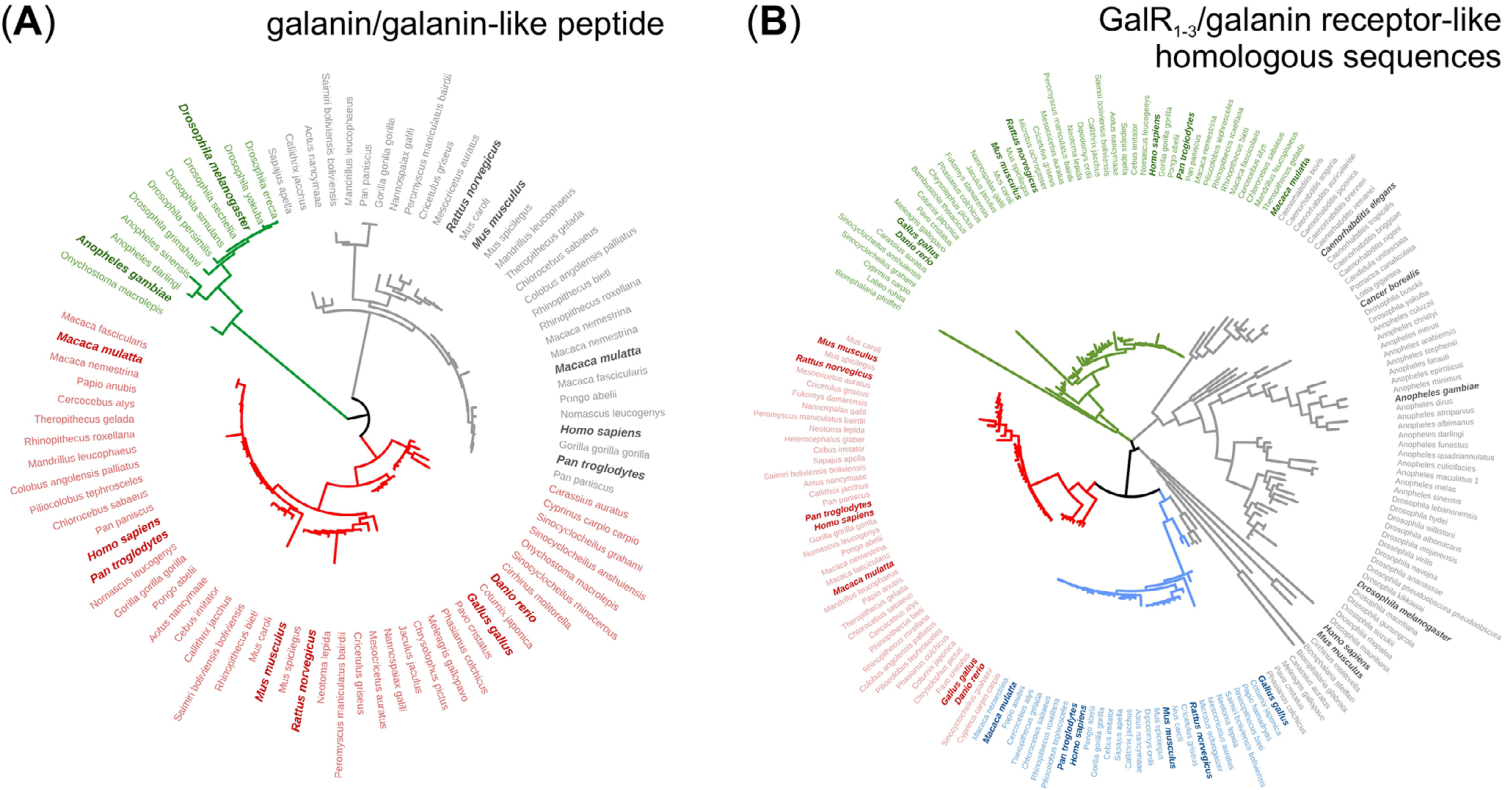
Phylogeny of galanin signaling in select categories of the animal kingdom. (A) UniProt data for galanin and peptides with significant sequence homology (6,231 in total; excluding spexin) were screened for the particular taxonomic categories that include model organisms frequently used in neuroscience research (in bold): *Caenorhabditis* genus (*Caenorhabditis elegans*), *Drosophila* genus (*Drosophila melanogaster*), *Anopheles* genus (*Anopheles gambiae*), *Cancer* genus (*Cancer borealis*), *Cyprinidae* family (*Danio rerio*), *Phasianidae* family (*Gallus gallus*), *Myomorpha* suborder (*Mus musculus, Rattus norvegicus*), and *Simiiformes* infraorder (*Homo sapiens, Pan troglodytes*, and *Macaca mulatta*). Sequences were saved in *FASTA*, processed in *Mega11* with maximum likelihood of tree construction, with circular phylogenic trees constructed in *iTol*. Taxa with galanin peptide appear in red, those with galanin‐like peptide are in gray, whereas alternative (uncharacterized) protein isoforms are in green. Taxa identified at more than one branch of the tree have multiple peptides (e.g., many mammals). (B) Galanin receptors (GalR1: green, GalR2: red, and GalR3: blue), as well as orthologs and presumed alternative receptors/homologous sequences (e.g., NPR‐9/galanin‐like G protein‐coupled receptor for *Caenorhabditis elegans*; allostatin receptor‐like protein for *Cancer borealis*; AstAR2G/G protein‐receptor family 1 profile domain‐containing protein for *Anopheles gambiae*; allostatin A receptor for *Drosophila melanogaster*; GPR151/galanin receptor‐like protein for both *Homo sapiens* and *Mus musculus*) were screened in 180 species as above. Note the many insect species likely using galanin‐like peptide‐based signaling systems, signifying both the early evolution and phylogenetic conservation of neuropeptide signaling. Many species harbor more than one GPCR for galanin binding.

Galanin, a 29/30‐AA neuropeptide, is one example for which remarkable phylogenetic conservation exists (Figure 1A), along with its receptor systems (Figure 1B). Alike for other neuropeptides, the larger phylogenetic distance between species, the higher the variability in the neuropeptide sequence, along with the existence of alternative and often uncharacterized receptor‐like sequences (Figure 1B).

The emergence of neuropeptides early in the animal kingdom greatly helped the elucidation of the diversity, homology, presence as well as functions of many neuropeptides in worms [13], slugs [14], insects [15], crabs [16], and other species. A series of seminal genetic and neurophysiology studies not only provided detailed anatomical diagrams but also clarified the use‐dependent production, signaling properties, developmental, and endocrine roles of many neuropeptides. These studies also highlighted many “translational” benefits, considering that invertebrate model organisms often offer better genetic access, faster rates of reproduction, shorter natural lifespan, and less complex neurocircuits than laboratory rodents. In this review, we emphasize mechanistic concepts for neuropeptides in the nervous systems of mammals particularly because of their unique developmental trajectories, cellular complexities, and the coexistence and cooperativity of fast neurotransmitter and neuropeptide systems.

## 3 Neuropeptide Metabolism: The Classical Concepts

**FIGURE 2:**
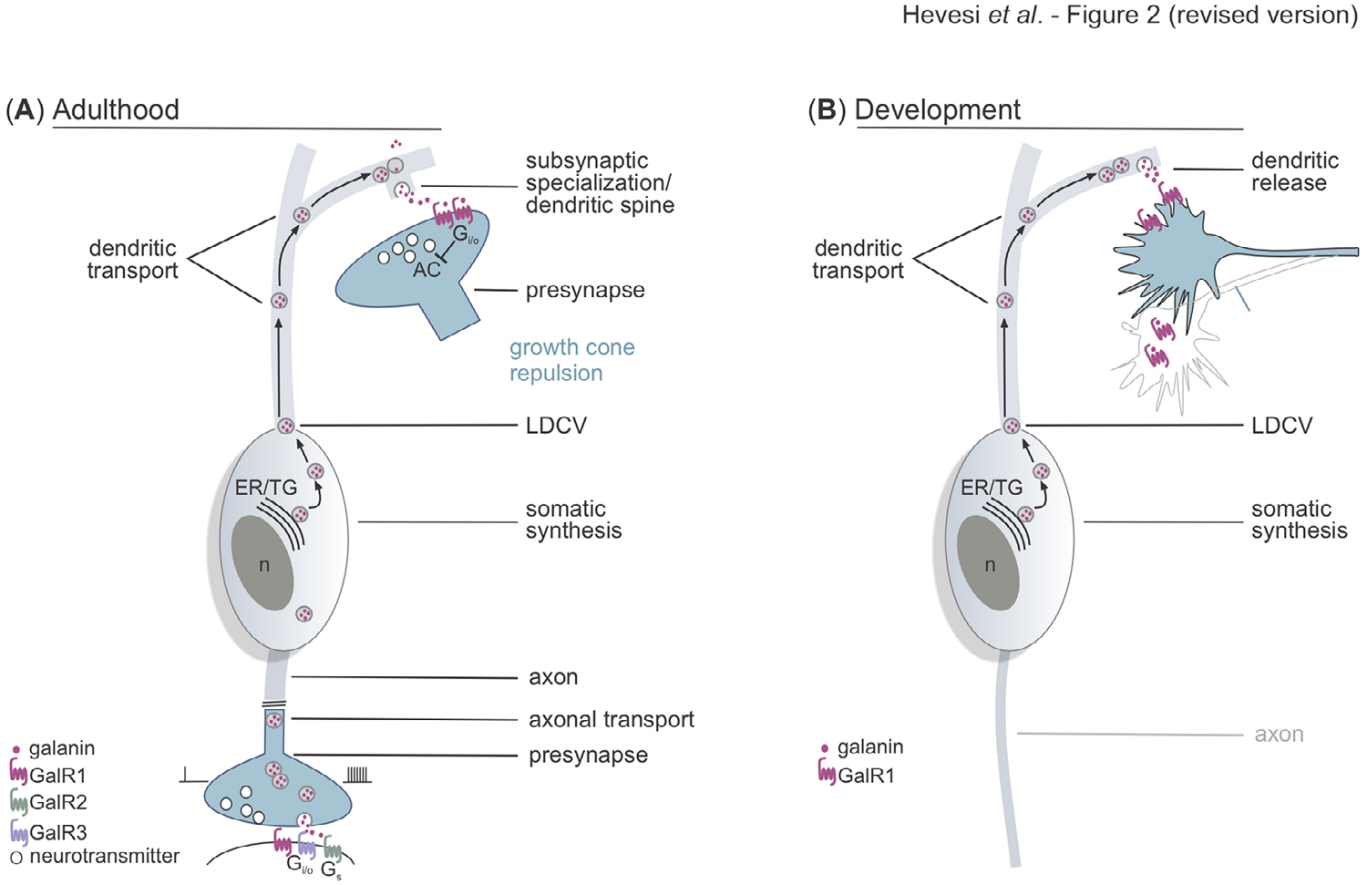
Neuropeptide signaling in adult and developing neuronal circuits. (A) In mature neurocircuits, neuropeptides can partake in both retrograde signaling at dendritic release sites and anterograde signaling from nerve terminals. In general, neuropeptides are thought to be released extrasynaptically. Similarly, neuropeptide GPCRs are considered to chiefly be inserted in the plasmalemma at perisynaptic locations. Retrograde release is thought to tune presynaptic release probability [43]. Anterograde neuropeptide release upon repetitive stimulation modulates the action of fast small‐molecule neurotransmitters. (B) During brain development, neuropeptides could participate in setting directional growth cone decisions when released from the dendrites of putative postsynaptic neurons. If neuropeptides are stimulatory, growth cones could be attracted, stabilized, and synapses formed. Upon repulsive guidance decisions, the trajectories of growth cones could be altered, and their motility slowed. ER/TG, endoplasmic reticulum/trans Golgi apparatus; GalR1‐3, galanin receptor 1–3 subtypes; LDCV, large dense‐core vesicle; n, nucleus.

The family of neuropeptides consists of short (3–30) amino acid (AA) sequences that serve as transmitter molecules [17], and are expressed virtually exclusively in neurons that signal via small molecule transmitters [18]. Despite being structurally heterogeneous, there are essential properties that neuropeptides share: they are synthesized as long, often multifunctional prepropeptides in the somatodendritic compartment of neurons, processed enzymatically to yield active peptides, which are then transported along the axons for storage in large dense‐core vesicles (LDCVs) prior to their activity‐dependent “quantal” release (Figure 2A; “*adult*”). Ribosomal neuropeptide synthesis takes time and is energy demanding. Likewise, the considerable distance between the place of neuropeptide biosynthesis (soma) and release (nerve endings) requires lengthy delivery times, even if anterograde LDCV trafficking toward synaptic terminals is fastest in neurons [19]. These long‐held views have been challenged relatively recently: Ludwig and Leng [20] showed neuropeptide release from dendrites, while Glock et al. [21] proposed neuropeptide synthesis in nerve endings. Thus, neuropeptide production, processing, and signaling in vertebrates fundamentally differ from the action of “fast” small molecule neurotransmitters, which are synthesized locally within axon terminals, stored in small/clear synaptic vesicles within and/or adjacent to the active zone of presynapses [22].

Action potential firing in neurons and the subsequent release of small‐molecule neurotransmitters engages ligand‐gated ion channels within the postsynapse, with metabotropic GPCRs accumulating extrasynaptically to mediate homeostatic signaling upon neurotransmitter spill‐over. In turn, neuropeptides were for decades thought to be released only upon high frequency neuronal firing or repetitive stimulation (>10 Hz), necessary to evacuate LDCVs [20, 23, 24]. Therefore, neuropeptide release events were thought to level out excess or errant neuronal firing to protect the structural integrity of neurocircuits. This notion was mechanistically supported by neuropeptides exclusively binding GPCRs with shallow inactivation kinetics and slow recycling [25].

Fast neurotransmitters have reuptake systems with transporter molecules eliminating excess ligands from the synaptic cleft [26]. The efficient recycling of neurotransmitters minimizes postsynaptic desensitization, thus ensuring millisecond‐scale precision of ligand availability in the synaptic cleft. In turn, neuropeptide degradation is an enzymatic process and occurs mostly in lysosomes. Thus, neuropeptides are “slow” neuromodulators with their actions being reliant on signal transduction cascades downstream of G proteins.

Galanin is produced by the cleavage of preprogalanin, which simultaneously generates galanin‐message associated peptide (59/60 AA), encoded at the 5′ end of the genomic galanin sequence [27]. Besides galanin and GMAP, members of the galanin neuropeptide family include galanin‐like peptide (GALP) (60 AA), spexin, and alarin (49 AA), produced by alternative splicing [28]. Galanin mRNA copy numbers/neuron are in the 10–50 molecule range at physiological “ground” states [29], with transcription induced (>100 fold) upon insults, such as pain and epilepsy [30, 31]. Galanin and galanin‐related peptides are transported in vesicles toward nerve endings for release upon action potential‐induced depolarization. Moreover, galanin has been localized to dendrites in neurons [32] where it could mediate the retrograde modulation of presynaptic neurotransmitter release once back‐propagating action potentials are produced  by a given neuron in the adult brain.

## 4 Neuropeptide Coexistence: A Paradigm Shift in Circuit Neurobiology

According to early interpretations of Dale's principle, each neuron shall contain a singular chemical messenger. This axiom was repeatedly challenged, for example, by demonstrating the co‐localization of small‐molecule transmitters [33] and then finding neuropeptide‐laden (cholecystokinin [CCK] and somatostatin) LDCVs in neurons synthesizing either norepinephrine [34] or dopamine [18, 35]. Based on these discoveries, the hypothesis of co‐released chemical messengers from some, if not all, nerve terminals has been proposed. Recently, single‐cell RNA‐sequencing corroborated that neither neurotransmitters nor neuropeptides exist alone [29], even in areas where the utilization of neuropeptides is thought to be “superior” to that of glutamate, dopamine, or GABA (e.g., hypothalamus). Thus, neurosecretory cells expressing a variety of peptides also express amino acid transmitters. As such, corticotropin‐releasing hormone (CRH)‐expressing neurons can signal via both GABA and glutamate [36] while magnocellular vasopressin and oxytocin‐positive neurons by glutamate [37]. Thus, it is an exciting development in circuit neuroscience how excitatory neuropeptide versus inhibitory neurotransmitter, excitatory neuropeptide versus excitatory neurotransmitter, and inhibitory neuropeptide versus inhibitory neurotransmitter (and other) pairs could configure circuit operations for context‐ and activity‐dependent behaviors [3].

For galanin, a primary nucleus of expression is the locus coeruleus [38, 39, 40], where most of the norepinephrine neurons express this neuropeptide. Likewise, some subsets of GABA (inter‐)neurons express galanin locally [41]. Notably, norepinephrine neurons co‐express, in addition to galanin, for example, *Carpt*, *Grp*, *Sst*, *Ucn*, and *Nps* mRNA transcripts [38], by which they can generate combinatorial codes through co‐existent receptor systems in the forebrain given the breadth of norepinephrine afferentation throughout. For developmental biology, large cohorts of GABA neurons transiently contain galanin [42]. In contrast, galanin released from glutamatergic neurons in the neonatal/juvenile thalamus contributes to the organization of sensory afferents for efficient neurotransmission in adult life, at least in mouse models [2]. This is made available by the complementarity of galanin receptor expression in those very afferents that innervate galanin‐rich territories [2].

## 5 Neuropeptide Release From Soma and Dendrites

It is by now broadly accepted that opposing signaling systems exist in virtually every synapse: the function‐defining neurotransmitter and some modulators are released anterogradely onto the subsynaptic dendrite to activate ligand‐gated intrasynaptic receptors, as well as peri‐ or extrasynaptic metabotropic receptors. In contrast, retrograde messengers (i.e., the fundamental regulatory value of the postsynaptic neuron to tune its inputs) rely on ligand synthesis and vesicular release from neuronal somata and dendrites [43]. Neuropeptides can also support retrograde signaling, a notion that has led to a sea‐change in viewing their action, when the dendritic release of vasopressin and oxytocin were identified in the hypothalamus and other sites [20, 44, 45]. As such, the distance between the site of neuropeptide synthesis/processing and release is shortened for dendritic release (Figure 2A), thus allowing faster ligand availability [45]. The speed of LDCV trafficking, regardless of its direction, is quasi‐equivalent in dendrites [19], allowing for the purposeful distribution of vesicles along release sites. Thus, neuropeptide action is local and confined to specific synapses, which contrasts “volume transmission” that relies on long‐range and spreading neuropeptide action [46]. Considering that neuropeptide synthesis is rapidly inducible, the above anatomical arrangement can help to fine‐tune neurocircuit operations upon (or in anticipation of) specific behaviors. Within a single synapse, neuropeptides can participate in both anterograde (presynaptic) and retrograde (somatodendritic) neurotransmission, likely allowing for combinatorial outcomes when more than one peptide is used at any point in time. This suggestion could explain why neuropeptides whose physiological sign can be “excitatory” but also “inhibitory”, dependent on the receptor system(s) available at the time (see below), are coincidently present within a single synapse: neuropeptides that evoke excitation likely sensitize to and/or lengthen anterograde neurotransmission, whereas peptides that activate inhibitory receptor systems could partake in the retrograde modulation of synaptic strength and plasticity. Lastly, neuropeptides, once released, induce long‐lasting modifications via GPCR engagement, thus changing membrane excitability, intracellular signaling, and even gene transcription: for example, CCK released from the dendrites of midbrain dopaminergic neurons modulates GABA inputs, thereby affecting both food reward and locomotion [47].

For galanin, its somatodendritic release can contribute to synapse selection, and circuit refinement in the whisker pathway during the first postnatal week, at least in laboratory rodents [2]. In established neurocircuits, dendritic galanin release can remain a critical feature, for example, in both norepinephrine‐containing and GABA neurons of the locus coeruleus [38, 40, 41], where it dampens synaptic signaling under physiological conditions. In pathobiology, increased *de novo* galanin expression is postulated to reduce circuit excitation in pain (analgesia) [48] and epilepsy (anticonvulsant action) [31], an observation also supported by the pharmacological modulation of galanin receptors in disease states and models. Thus, neuropeptides not only coexist but cross‐modulate fast neurotransmitter action through GPCR signaling.

## 6 Neuropeptide Receptors: GPCRs With Many Modalities

Receptor diversity in space and time is the chief molecular determinant of any chemical messenger's action. For fast neurotransmitters acting at orthostatic as well as allosteric binding sites of ligand‐gated channels, variations in receptor kinetics and localization are produced by the combinatorial recruitment of their subunits. For neuropeptides, structural differences in GPCR subtypes diversify G protein recruitment, internalization kinetics (e.g., by β‐arrestins), and the binding of regulatory proteins at their C‐termini [25]. Thus, receptor activation is usually prolonged, allowing for cell state‐changes to occur and impact both neurocircuit architecture and output (particularly, cellular and tissue‐wide metabolism).

GPCRs are the largest superfamily of transmembrane receptors, and their functional diversity appeals to drug development. Nevertheless, neuropeptide research is hindered by evolutionary variations of neuropeptide receptor expression (Figure 1B), identity, and signal competence (Figure 1A,B; for galanin see also Ref. [49]). The propensity of GPCRs to form homo‐ or heterooligomers and ensuing changes in signal transduction (e.g., G protein switching [50]), can produce unexpected and even unwanted off‐target effects. It is particularly concerning that small‐molecule and blood‐brain‐barrier penetrating ligands for neuropeptide GPCRs often show serious side effects, in particular liver toxicity. Nevertheless, FDA‐approved drugs acting via peptidergic mechanisms are now available to treat, for example, migraine, insomnia, diabetes, and obesity. The status quo may further improve through extensive data from single‐cell RNA‐sequencing studies [29, 38, 42, 51], and because of the many new optical tools (e.g., G protein activation‐based sensors) and genetically engineered (in‐)vertebrate models that have become available [52].

For galanin, its binding to three receptors (GalR_1_, GalR_2_, and GalR_3_) has been verified [27, 53]. Nevertheless, galanin fragments (e.g., 1–16, 2–11, 2–29), GMAP, alarin, and GALP all bind GalR_1–3_ with varying affinities [27]. GalR_1_ and GalR_3_ signal through G_i/o_ proteins, whereas GalR_2_ recruits G_s_ proteins. Galanin receptors can heteromerize with, among others, 5‐HT_1A_, neuropeptide Y (NPY) receptor Y_1_, and α_2_‐adrenoreceptors, and even trimerize when GalR_1_ and GalR_2_ co‐exist with another receptor [50]. Therefore, G_i/o_ protein‐coupled GalR_1_ evokes pro‐depressant and anxiogenic effects through inhibitory signal transduction. Conversely, GalR_2_ activation and G_q/11_ protein recruitment [53] is antidepressant and anxiolytic. For neuronal development and circuit operations, GalR_1_‐mediated signaling can induce axonal growth cone repulsion [54], synapse selection [2], and synaptic depression (e.g., in epilepsy) [31]. In contrast, GalR_2_‐dependent signaling could promote cell cycle progression and proliferation.

## 7 Transient vs. Persistent Expression Sites for Neuropeptides in Circuit Development and Operations

A unique feature of many neuropeptides is that they can have transient expression sites, which are often considered as respective foci of fate progression and/or neural activity during development and in adulthood, respectively [2]. Early on, Tohyama's team [55] has studied the ontogeny of many neuropeptides/neuroactive substances in the rat central nervous system [56]. Their results suggest at least three types of developmental expression patterns, often within a brain region: (i) neuropeptides appear in the prenatal period with their levels gradually increasing until reaching a plateau in the early postnatal period. Subsequently, neuropeptide expression is maintained or slightly reduced in adulthood. Substances P, CCK, and NPY are known examples. (ii) Neuropeptides first appear perinatally, peak shortly, followed by a drastic reduction. Somatostatin (SST), CCK, NPY, neuropeptide W (NPW), proopiomelanocortin, and galanin in some subsystems belong to this group [2, 57–59]. These often “uncharacteristic” or “elusive” neuropeptide expression patterns during brain development suggest that neuropeptides are likely more than sophisticated markers of some neurons. Instead, neuropeptides could be associated with bouts of neuronal activity during specific stages of neurocircuit maturation. (iii) Neuropeptides first appear in the late prenatal stage or after birth and reach their maximum in juvenile animals (examples are vasopressin, oxytocin, vasoactive peptide [VIP], and galanin). In case of cascading expression across brain areas, one can postulate that differences in the timing of circuit maturation could account for the differential timing of neuropeptide expression. Accordingly, neuropeptides could mark a conserved function, be this excitation, inhibition, or modulation of excitation (considering that GABA is seen as excitatory during development). Another open question is if neuropeptides are redundant; that is, not their molecular identity, but rather cumulative action on GPCRs and the ensuing “signaling tone” (G_s/q_ *vs*. G_i/o_), which is essential to entrain a specific neurocircuit. Thus, neuropeptides could be critical for synapse selection, maturation, and maintenance.

For galanin, transient somatodendritic expression (Figure 2B) was detected in glutamatergic neurons of the ventrobasal thalamus (VB) [2], a brain area that serves as the main subcortical relay in the sensory whisker pathway. Once released from the dendrites of VB neurons, galanin acts on central axons emanating from the brainstem's trigeminal nucleus, which express GalR_1_. In vitro data had earlier shown that GalR_1_ activation on axons induces growth cone repulsion [54], a mechanism compatible with the activation of G_i/o_ protein‐coupled GPCRs during the directional turning of axonal growth cones [60, 61]. Thus, the net effect of galanin‐GalR_1_ signaling could contribute to the refinement of sensory connectivity at the thalamic level, whose impairment might evoke life‐long behavioral maladaptation. Notably, galanin is only expressed between embryonic day 18 and postnatal day 14 in the VB, coincident with circuit maturation, but not in adulthood. This observation reinforces that galanin signaling might underpin a temporally controlled event of neurocircuit maturation.

## 8 Conclusions and Future Perspective

Neuropeptides are fundamental to the structure and function of the nervous system. Recent insights suggest that neuropeptide signaling was sufficient for evolutionarily early, simpler nervous systems to operate as nets of peptidergic cells [8, 11]. During evolution in the animal kingdom, the role of neuropeptides became diversified and refined. It is broadly accepted that synapses commonly use neuropeptides to modulate the action of fast, primary neurotransmitters, and even execute intercellular communication on their own along prolonged timescales. Given that neuropeptide genes are highly inducible, transient foci for their expression are being recognized. This notion is conceptually significant because neuropeptides can gain greater appreciation as signaling molecules during brain development, particularly for neural progenitor propagation, cell migration, and circuit wiring. The many GPCRs that neuropeptides can engage provide sophisticated and cell‐state‐specific hubs for signaling. Among the exciting questions a greater understanding of the epigenetic regulation of neuropeptide expression, particularly in transgenerational settings or congenital diseases, could be leveraged to interrogate the maintenance versus elimination, and even positioning of synapses. Using evolutionary comparisons will be important to increase the translational value of experimental findings. For discovery research, causality between neuropeptide signaling and behavioral states [3] could advance insights into neurocircuit designs, synaptic plasticity, and operational flexibility of the mammalian nervous system. Notably, neuropeptide expression is sensitive to exposure to both illicit and prescribed psychoactive drugs [62], with altered neuropeptide levels seen in, for example, autism spectrum disorder and schizophrenia. Therefore, studying how either antenatal or adolescent drug exposure could change the neuropeptide landscape of specific cell types, neuronal circuits, or even brain areas could offer valuable insights in mechanisms of vulnerability in addiction pathobiology. Thus, neuropeptides are fundamental mechanistic players in the nervous system, with future discoveries poised to decipher how their impact on neurocircuit formation could evoke behavioral state‐changes throughout the lifespan, and also identify critical vulnerabilities for neurodevelopmental disorders.

## Author Contributions

Z.H., T.Hö., and T.Ha. defined the scope of the study. Z.H. performed literature search and drafted the manuscript. T.Hö. and T.Ha. proofread and commented on earlier versions of this review.

## Conflicts of Interest

The authors declare no conflicts of interest.

## Data Availability

Data sharing are not applicable to this article as no datasets were generated or analyzed in‐depth during either the conceptualization or the writing of the current review. Data to construct phylogenetic trees are available in UniProt (uniprot.org).
